# A Very Bright Far-Red Bioluminescence Emitting Combination Based on Engineered Railroad Worm Luciferase and 6′-Amino-Analogs for Bioimaging Purposes

**DOI:** 10.3390/ijms22010303

**Published:** 2020-12-30

**Authors:** Vadim R. Viviani, Vanessa R. Bevilaqua, Daniel R. de Souza, Gabriel F. Pelentir, Michio Kakiuchi, Takashi Hirano

**Affiliations:** 1 Graduate Program of Evolutive Genetics and Molecular Biology, Federal University of São Carlos (UFSCar), 18052-780 São Carlos, São Paulo, Brazil; vani_bevilaqua8@hotmail.com; 2 Graduate Program of Biotechnology and Environmental Monitoring, Federal University of São Carlos (UFSCar), 18119-001 Sorocaba, São Paulo, Brazil; phor.souza@gmail.com; 3 Department of Physics, Chemistry and Mathematics, Federal University of São Carlos (UFSCar), 18052-780 São Carlos, São Paulo, Brazil; gabrielpelentir@yahoo.com.br; 4 Department of Engineering Science, Graduate School of Informatics and Engineering, The University of Electro-Communications, Chofu, Tokyo 182-8585, Japan; k1533023@edu.cc.uec.ac.jp (M.K.); thirano@uec.ac.jp (T.H.)

**Keywords:** bioimaging, Far-Red bioluminescence, NIR bioluminescence, luciferin amino-analogs, biophotonics

## Abstract

Beetle luciferases produce bioluminescence (BL) colors ranging from green to red, having been extensively used for many bioanalytical purposes, including bioimaging of pathogen infections and metastasis proliferation in living animal models and cell culture. For bioimaging purposes in mammalian tissues, red bioluminescence is preferred, due to the lower self-absorption of light at longer wavelengths by hemoglobin, myoglobin and melanin. Red bioluminescence is naturally produced only by *Phrixothrix hirtus* railroad worm luciferase (PxRE), and by some engineered beetle luciferases. However, Far-Red (FR) and Near-Infrared (NIR) bioluminescence is best suited for bioimaging in mammalian tissues due to its higher penetrability. Although some FR and NIR emitting luciferin analogs have been already developed, they usually emit much lower bioluminescence activity when compared to the original luciferin-luciferases. Using site-directed mutagenesis of PxRE luciferase in combination with 6′-modified amino-luciferin analogs, we finally selected novel FR combinations displaying BL ranging from 636–655 nm. Among them, the combination of PxRE-R215K mutant with 6′-(1-pyrrolidinyl)luciferin proved to be the best combination, displaying the highest BL activity with a catalytic efficiency ~2.5 times higher than the combination with native firefly luciferin, producing the second most FR-shifted bioluminescence (650 nm), being several orders of magnitude brighter than commercial *AkaLumine* with firefly luciferase. Such combination also showed higher thermostability, slower BL decay time and better penetrability across bacterial cell membranes, resulting in ~3 times higher in vivo BL activity in bacterial cells than with firefly luciferin. Overall, this is the brightest FR emitting combination ever reported, and is very promising for bioimaging purposes in mammalian tissues.

## 1. Introduction

Bioluminescence, the emission of visible light for communicative purposes by living organisms, has been also extensively used for bioanalytical purposes [1,2]. Bioluminescence is emitted in a wide range of colors ranging from violet to red depending on the organism [3]. Whereas the most common colors are blue for marine organisms, and green-yellow for terrestrial ones, red bioluminescence is very rare, being found exclusively in two marine species and in *Phrixotrix* spp. railroad worms [3].

In recent years, red bioluminescent emitting systems are gaining much interest for bioimaging of biological and pathological processes in mammalian organisms [1,2], including metastasis proliferation and tracking bacterial and viral infections, including SARS-covids [4]. Firefly and other beetle luciferases have been the most-used reporters, because these luciferases elicit light emission in the green-red region of the spectrum with high quantum yield [5,6], using the same biochemical reaction: the ATP-dependent oxidation of a benzotiazolic luciferin.

To extend the applicability of beetle luciferases for bioimaging purposes, novel luciferases were cloned in the past few decades [7,8,9,10,11,12,13,14,15,16,17,18,19,20]. To date, the only luciferase that naturally produces red light is still that of *Phrixotrix hirtus* railroad worm (PxRE), with an emission peak at 623 nm [15]. The luciferases of *Photinus pyralis*, *Luciola cruciata*, *L. mingrelica* fireflies and *Pyrophorus plagiophtalamus* click beetles have also been engineered to produce red light [20,21,22]. However, all these wild-type and modified luciferases still elicit BL spectra at shorter wavelengths (<620 nm) than ideally required for bioimaging purposes in mammalian tissues, which are rich in hemoglobin, myoglobin and melanin, that strongly absorb in the blue-orange range of the spectrum. Unfortunately, as pointed out by K. Wood in the 1990s (1991) [22], there is an upper limit of ~640 nm to which BL spectra can not be further red-shifted by simply engineering beetle luciferases, which essentially depends on the structure and spectroscopic properties of natural firefly d-luciferin (LH_2_) and its product, oxyluciferin. In practice, no one ever succeeded in engineering an efficient luciferase emitting at a wavelength peak longer than 625 nm.

Far-Red (FR) and Near-Infrared (NIR) emitting luciferases would be better suited for such purposes, due to the better penetrability of light at these wavelengths in mammalian tissues. Several alternatives to produce FR and NIR emitting systems have been used [23]. Branchini et al. (2010) [24] and Kojima et al. [25] attached fluorophores to firefly luciferase structure which, based on the BRET principle of energy transfer from excited luciferase-oxyluciferin complex to the fluorescent acceptor, emit NIR bioluminescence. However, such systems have the drawback that they need chemical modification and are not practical for in vivo gene reporting.

Another approach was the chemical synthesis of novel FR and NIR emitting luciferin analogs. Several luciferin analogs have been already synthesized [26,27,28,29], including 6′-aminoluciferin [26,27], blue light emitting analogs [28,29], and several modified amino-analogs producing light in the orange-red region with firefly luciferases [30,31,32].

More recently, some FR and NIR analogs were synthesized. Among them, *AkaLumine*, which is a luciferin analog with an extended π-electronic conjugation system which emit at 675 nm with firefly luciferase (FF-Luc) [33], and its more soluble HCl salt (called as TokiOni), which emits at 677 nm with FF-Luc and displays much higher penetrability in mammalian cells [34] are commercially available. However, these FR and NIR analogs still suffer of lower bioluminescence activity with native firefly luciferases in relation to LH_2_.

In order to obtain more active FR and NIR bioluminescence emitting systems, luciferases better suited for such FR and NIR emitting analogs were also engineered. These systems include: (AkaLuc) a mutant luciferase specifically designed to optimize the BL performance with AkaLumine [35], which emits above 650 nm with 7 times higher BL activity than the original firefly luciferase with AkaLumine, keeping an activity of ~1.3% of that of FF-Luc with LH_2_; (5-allyl-6-dimethylamino-2-naphthylethenyl luciferin), which emits at 705 nm with firefly luciferase and at 655 nm with the modified AkaLuc [36]; (Infraluciferin), which has an extended conjugation system that emits with maximum wavelength at 670 nm with firefly luciferase, at 706 nm with S284T red-emitting mutant, and at 655 nm with X5FF-Luc [37]. Using Infra-luciferin and two mutant firefly luciferases, Stowe et al. demonstrated the feasibility of dual bioluminescence imaging in NIR region in mouse cancer models [38]; (naphthyl-luciferin) with mutant click-beetle luciferase, which emit at 730 nm [39]. Despite the improved properties, all these FR and NIR emitting combinations still suffer of lower activity when compared with the combination of native luciferin with wild-type and engineered luciferases.

The red-emitting luciferase of *Phrixotrix hirtus* railroad worm could be a good starting point to develop far red-shifted emitting luciferases. However, previous site-directed mutagenesis studies failed to considerably change the bioluminescence color [40]. Most mutations did not affect the color and decreased the activity. The only mutations that changed the bioluminescence spectrum caused 10–15 nm blue-shifts and were located close to the bottom of the luciferin binding site, giving important insights on critical regions of the active site for bioluminescence spectra modulation [41]. Noteworthy, we found that PxRE red-emitting luciferase in the presence of larger cyclic 6′-aminoluciferin-analogs, display more red-shifted spectra with considerably higher bioluminescence activity when compared to green-yellow emitting beetle luciferases with the same substrates [42,43]. Among the analogs tested, 6′-pyrrolidinylluciferin was the substrate which usually gave highest activity. Modelling and site-directed mutagenesis studies showed that such higher activities were due to a larger oxyluciferin phenolate binding site in this luciferase, which can better accommodate larger 6′-substituted amino-analogs [43].

Based on the above knowledge, we used PxRE luciferase site-directed mutagenesis in combination with distinct 6′-amino-analogs to screen novel more efficient FR emitting systems. Using such an approach, we developed a novel much brighter FR emitting combination with evident applicability for bioimaging in mammalian tissues.

## 2. Results and Discussion

### 2.1. Background

**Figure 1:**
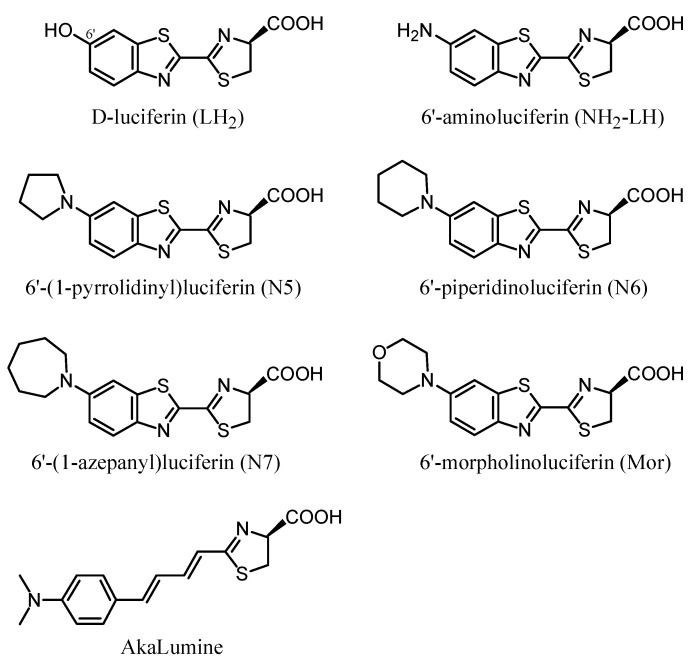
Firefly d-luciferin (LH_2_) and its analogs.

Previously, we reported that PxRE displays much higher bioluminescence activity and more red-shifted spectra with 6′-amino-analogs than any other green-yellow emitting luciferase [42,43]. This higher activity was shown to be due to a larger phenolate oxyluciferin binding site, which can better accommodate larger 6′-substituted amino-analogs. Among the analogs previously tested, the 6′-pyrrolidinylluciferin (N5) displayed the highest bioluminescence activity with PxRE luciferase [43]. Thus, in order to find mutant luciferases with improved activity and more far-red-shifted spectra, here we made and screened mutants of PxRE luciferase with 6′-pyrrolidinylluciferin (N5), and investigated the bioluminescence properties with this analog, with 6′-aminoluciferin (NH_2_-LH) and other 6′-substituted-amino-analogs displaying different sizes, including those with rings ranging from 5 to 7 atoms [piperidino (N6), 1-azepanyl (N7) and morpholino (Mor)], and with the FR emitting commercial analog, *AkaLumine* (Figure 1).

### 2.2. FR-Emitting Mutant Selection

**Figure 2:**
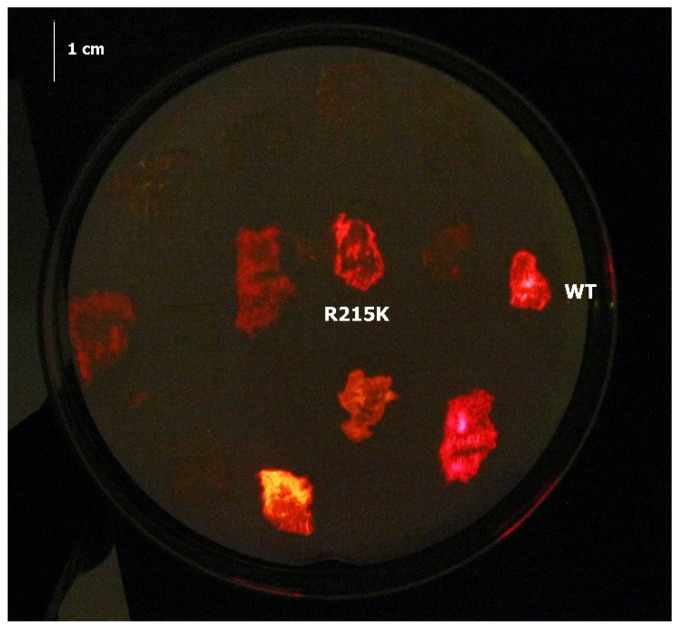
Selection of *Phrixotrix hirtus* luciferase FR mutants using 6′-(1-pyrrolidinyl)luciferin (N5).

We used previously made PxRE luciferase mutants (H209A, C311T, L334R, L246M, S220R, A246G, S314T, H242K), and prepared other single point mutants (L348V and L348A) for screening their bioluminescence (BL) activity and color with 6′-(1-pyrrolidinyl)luciferin (N5), and compared with the wild-type enzyme. Among the *E. coli* colonies expressing luciferase mutants, RE-R215K showed highest in vivo BL activity when compared to colonies expressing the wild-type enzyme and other mutants (Figure 2). We then selected this mutant and characterized its bioluminescence properties, and compared with other luciferin analogs. The mutant R215K stood out for its much higher bioluminescence activity in relation to other analogs and luciferin, and in relation to the wild-type luciferase, and for its further red-shifted spectrum.

### 2.3. BL Properties of RE-R215K with Firefly Luciferin

Table 1 summarizes the kinetic and spectral bioluminescence (BL) properties of WT and mutant RE-R215K luciferases. Both the specific bioluminescence activities with LH_2_ and ATP, or with luciferyl-adenylate, were slightly higher for this mutant in relation to those of the wild-type (WT) enzyme with the same substrates. As expected, both the overall and oxidative catalytic constants were also slightly higher for the mutant in relation to the WT enzyme. The K_M_ value for luciferin increased considerably (~4 times) for this mutant, indicating decrease in affinity for the natural substrate, whereas that for ATP decreased almost four times. On the other hand, whereas the catalytic efficiency regarding luciferin was slightly lower for the RE-R215K mutant, that for ATP was almost 5 times higher than for the WT enzyme. The bioluminescence spectrum of R215K mutant with luciferin (629 nm) luciferase also slightly changed, being ~6 nm red-shifted in relation to that of the WT enzyme.

### 2.4. Bioluminescence Properties of R215K with 6′-Amino-Analogs and Akalumine

#### 2.4.1. Bioluminescence Activity

**Figure 3:**
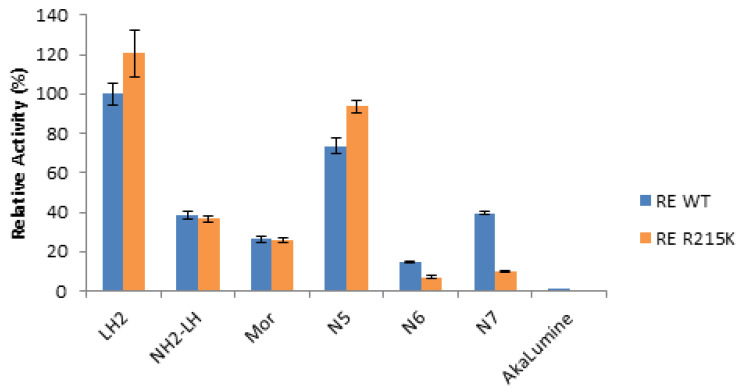
Comparison of the bioluminescence activity of *P. hirtus* Wild-type (WT) and R215K mutant luciferases with luciferin (LH_2_), 6′-amino luciferin analogs and commercial AkaLumine, at the final concentration of 1 mM. The results were normalized for *P. hirtus* wild-type luciferase activity with LH_2_. The standard deviations of activity averaged 3.15% and ranged from 0.3 to 11.9%.

**Figure 4:**
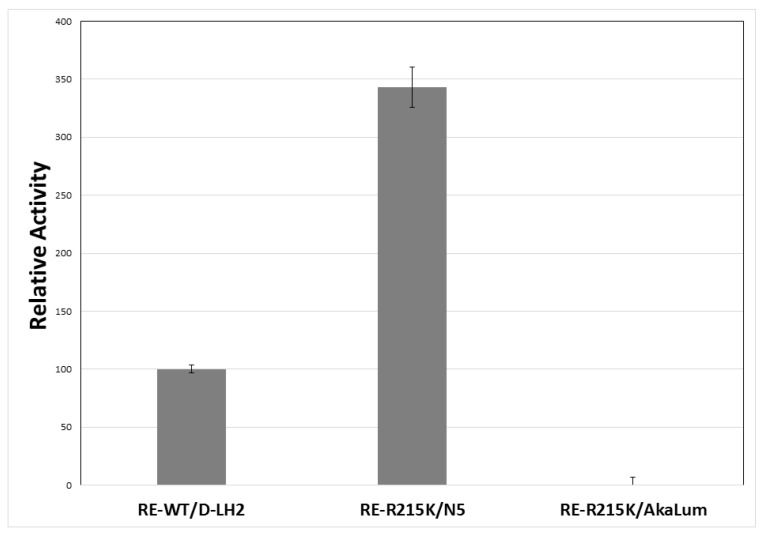
Comparison of the bioluminescence activity of *P. hirtus* wild-type (RE-WT) and R215K mutant luciferases with 100 µM d-luciferin (d-LH_2_), 6′-(1-pyrrolidinyl)luciferin (N5) and commercial AkaLumine (AkaLum). The results were normalized for *P. hirtus* wild-type luciferase activity with LH_2_. The standard deviations of activity averaged 3.15% and ranged from 0.3% to 11.9%.

We then compared the bioluminescence (BL) activity of different luciferin analogs with the mutant R215K (Figure 3). Among all the 6′-amino-analogs, N5 at the concentration of 1 mM stood out for its considerably higher activity, showing slightly higher activity than WT luciferase with the same substrate, and almost the same activity of 1 mM LH_2_ (Figure 4; Table 2). However, at the concentration of 100 µM, the activity of N5 with R215K mutant luciferase was nearly 3.5 times higher than that with d-luciferin at the same concentration (Figure 4), due to the much lower K_M_ value for N5. On the other hand, the commercial *Akalumine-HCl* luciferin analog gave orders of magnitude lower activity with both wild-type and R215K mutant luciferases.

#### 2.4.2. Substrate K_M_ and Luminescence Kinetics

**Figure 5:**
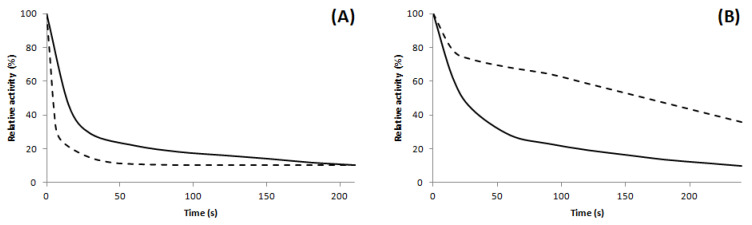
Kinetics of luminescence reaction of *P. hirtus* luciferases: (**A**) wild-type luciferase; (**B**) RE-R215K mutant. (full line) LH_2_; (dotted line) N5.

Previously, we reported that 6′-amino-luciferin analog displays much lower K_M_ value than LH_2_ with *P. hirtus* luciferase. Similarly, the K_M_ for 6′-(1-pyrrolidinyl) analog N5 (0.5 µM) was 14 times lower than that of natural LH_2_ (7 µM) for the wild-type luciferase, and 40 times lower (LH2: 40 µM and N5: 1 µM) for the mutant RE-R215K luciferase (Table 2), indicating that N5 is much better substrate for the R215K mutant luciferase than natural luciferin. As a consequence, the mutant enzyme saturates at much lower concentration of N5 analog. The kinetics of the luminescence reaction with N5 also became slower in relation to that of LH_2_ (Figure 5), with a half-life of ~180 s instead of 10 s with LH_2_.

#### 2.4.3. Catalytic Constant and Efficiencies

The catalytic constants of wild-type and RE-R215K mutant luciferases for luciferin (LH_2_) and N5 were similar to each other (Table 1). However, the whereas catalytic efficiencies of WT and R215K luciferases are ~12 and ~8 times higher for N5 in relation to LH_2_, respectively, the catalytic efficiency of R215K luciferase with N5 is ~40 times more efficient than wild-type enzyme with LH_2_.

#### 2.4.4. Bioluminescence Spectra

**Figure 6:**
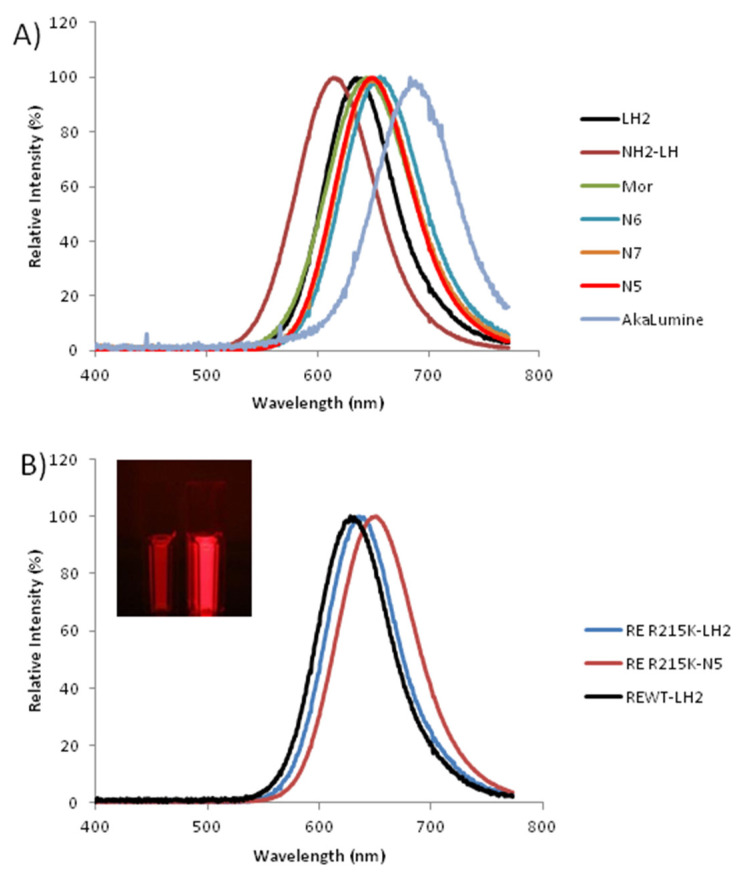
Normalized bioluminescence spectra of: (**A**) RE-R215K luciferase with LH_2_ and 6′-amino-analogs; (**B**) *P. hirtus* WT and R215K mutant luciferases with LH_2_, N5 and AkaLumine; (Upper panel in B) in vitro red bioluminescence of wild-type and R215K mutant luciferases with 0.1 mM LH_2_ and N5.

Figure 6 and Table 2 show the bioluminescence spectra and spectral properties of WT and mutant luciferases with luciferin and 6′-amino-analogs. Among all analogs tested, N5 (650 nm) and N7 displayed the most red-shifted spectra (655 nm) (Figure 6). The spectra were 31–33 nm blue-shifted only with regard to that of the commercial analog, *AkaLumine* (681–683 nm).

#### 2.4.5. Thermostability

**Figure 7:**
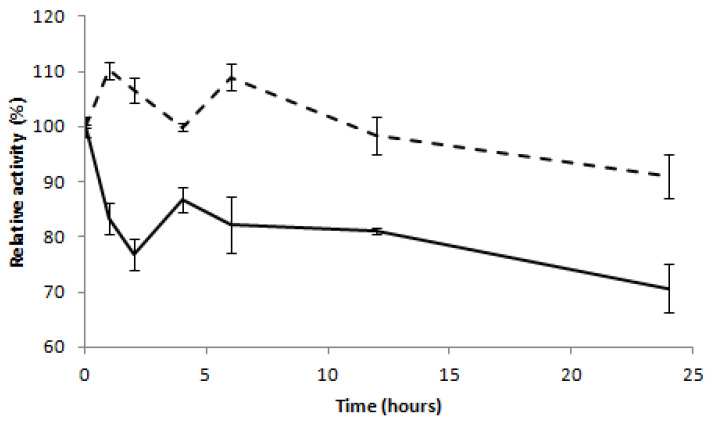
Thermostability of *Phrixotrix hirtus* red emitting luciferase (bold line) and its R215K mutant (dashed line). The standard deviations of activity averaged 2.4% and ranged from 0.3% to 5%.

We also compared the thermostability of R215K mutant with the wild-type enzyme (Figure 7). The mutant luciferase displayed slightly improved thermostability, retaining ~91% activity when incubated at 22 °C during 24 h, in comparison with the wild-type enzyme that retained only ~70%.

### 2.5. Bioluminescence of Bacteria Expressing R215K with 6′-(1-pyrrolidinyl)luciferin

**Figure 8:**
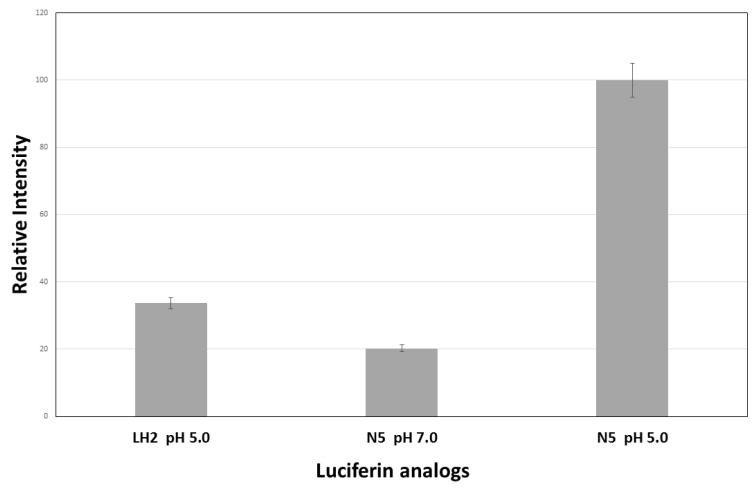
Comparison of in vivo bioluminescence activity of *E. coli* bacteria expressing PxRE-R215K luciferase upon addition of 1 mM LH_2_ at pH 5.0 and N5 at pH 7.0 and 5.0. d-luciferin and N5 analog are usually provided at acidic pH (5.0) to allow better penetrability across cell membranes.

In order to check the cell membrane penetrability and in vivo luminescence properties of such analogs, we tested the in vivo BL activity of *E. coli* liquid cultures after mixing LH_2_ and the N5 at neutral and acidic (pH = 5.0) pH (Figure 8). At 1 mM concentration and at pH~7.0, when the membrane permeability is expected to be lower due to the ionized substrate carboxyl group, N5 gave almost the same in vivo bioluminescence activity of LH_2_ at pH 5.0. However, when provided at pH 5.0, N5 showed about three times higher bioluminescent activity than LH_2_ at the same pH and concentration. These results show that N5 has much better penetrability across the biological membranes, and therefore is a better substrate for bioimaging. Additionally, the in vivo bacterial bioluminescence with N5 showed a more sustained kinetics, with a half-life of 33 min, in relation to LH_2_ which showed a half-life of 20 min (Figure S1 Supplemental information).

### 2.6. Structure and Function Relationships

The residue R215 (R218 in *P. pyralis* firefly luciferase) is an invariable residue in beetle luciferases, which is located at the bottom of luciferin binding site, close to oxyluciferin phenolate [43]. This arginine guanidinium group has been claimed to be important for bioluminescence spectra, since its mutation in yellow-green emitting firefly luciferase and in *P. vivianii* railroad worm green emitting luciferase promoted red-shifts [44,45]. In *P. hirtus* red emitting luciferase, however, the mutation of this residue, had almost no effect on the spectrum [45]. It was suggested, and later confirmed [42], that in PxRE luciferase this residue side-chain is spatially displaced from oxyluciferin phenolate, in relation to the closer green emitting *P. vivianii* luciferase. Whereas the mutation R215K, performed in this work, did not changed the electrostatic environment around excited oxyluciferin phenolate, as it preserves the positive charge, the shorter side-chain of lysine may contribute to increase the size of the cavity and the distance of the side-chain in relation to oxyluciferin phenolate group, explaining the accommodation of the larger substituted 6′-amino-analogs resulting in higher substrate affinity and activities, and red-shifted spectra.

### 2.7. Comparison RE-R215K/N5 with Other Beetle FR Emitting Luciferin-Luciferases

Overall, the above results clearly show that the combination of *P. hirtus* R215K mutant luciferase with N5 is the brightest FR emitting combination ever reported. Even if this combination is blue-shifted in relation to the commercially provided *AkaLumine* with firefly luciferase and other reported NIR emitting analogs (>700 nm), its much higher bioluminescence activity and catalytic efficiency may compensate the slightly lower tissue penetrability of bioluminescence at these more red-shifted wavelengths. Furthermore, the higher penetrability of N5 across cell membranes, the higher life time of in vivo bioluminescence, the much lower substrate requirement in in vivo assays using N5, and the higher thermostability of R215K luciferase in relation to the wild-type PxRE enzyme, make this combination a very promising tool for deep tissue bioimaging in mammalian organisms.

## 3. Material and Methods

### 3.1. Plasmids and Beetle Luciferases cDNAs

The cDNAs for *Phrixotrix hirtus* red-emitting (PxRE)**, was previously subcloned into pCold-vector (Takara). The mutants C311T, H242K, L334R, L246M, S220R, A246G, S314T were previously obtained. Commercial *P. pyralis* firefly luciferase was purchased from Promega (Madison, WI, USA).

### 3.2. Site-Directed Mutagenesis

The mutants R215K were obtained by site-directed mutagenesis using a ThermoFisher Scientific kit (Catalog F-530XL and F530L). The plasmids containing the luciferase cDNAs were amplified using *Phusion* polymerase and 2 complementary *primers* containing the desired mutation, using a thermal cycler (1 cycle 98 °C, 2 min; 25 cycles 98 °C, 30 s; 55 °C, 1 min and 72 °C, 6 min). After amplification, mutated plasmids containing staggered nicks were generated. The products were treated with *Dpn* I in order to digest non-mutated parental plasmids, and used directly to transform *E. coli* XL1-Blue cells (Agilent, Santa Clara, CA, USA). The following forward and respective reverse primers were used (the mutation codon is highlighted in bold): RE-R215K forward: GCA TCA CCA TCA AAT TCG TCC ACA GC and Reverse: GCT GTGGACGAATTTGATGGTGATGC.

### 3.3. Luciferase Expression and Purification

For luciferase expression, transformed *E. coli* BL21-DE3 cells were grown in 100–1000 mL of LB medium at 37 °C up to OD600 = 0.4, and then induced at 18 °C with 0.4 mM IPTG during 18 h. Cells were harvested by centrifugation at 2500× *g* for 15 min and resuspended in extraction buffer consisting of 0.10 M sodium phosphate buffer, 1 mM EDTA, 1 mM DTT and 1% Triton X-100, 10% glycerol and protease inhibitor cocktail (Roche), pH 8.0, lysed by ultrasonication and centrifuged at 15,000× *g* for 15 min at 4 °C. The N-terminal histidine-tagged PxRE, R215K luciferases were further purified by agarose-Nickel affinity chromatography (QIAGEN), using the following buffers: (Wash buffer) 50 mM Phosphate pH 7.0; 300 mM NaCl, 20 mM Imidazole; (Elution buffer) 50 mM Phosphate pH = 7.0; 300 mM NaCl, 250 mM Imidazole) and (dialysis buffer) 25 mM Tris-HCl pH = 8.0, 10 mM NaCl, 1 mM EDTA, 2 mM DTT, and 10% glycerol). The concentrations of purified luciferases were between 0.5 and 1 mg/mL, and the estimated purity, according to SDS-PAGE gels were about 90%.

### 3.4. Measurement of Luciferase Activity

Luciferase bioluminescence intensities were measured using an AB2200 (ATTO; Tokyo, Japan) luminometer. The assays were performed by mixing 5 µL of 40 mM ATP/80 mM MgSO_4_ with a solution consisting of 5 µL of luciferase and 85 µL of 0.5 mM luciferin in 0.10 M Tris-HCl pH = 8.0 at 22 °C. All measurements were done in triplicate for at least three independent luciferase preparations, and the averages and the standard deviations were reported in the figures.

### 3.5. Kinetics Measurements and K_M_ Determination

The K_M_ assays for luciferin were performed by mixing 5 μL of 40 mM ATP, 80 mM MgSO_4_ in a solution containing 10 μL of luciferase, 85 μL of 0.10 M Tris-HCl (pH 8.0) and luciferin and N5 at final concentrations between 0.01 and 1 mM. The K_M_ assays for ATP were performed by mixing 5 μL of 80 mM MgSO_4_ in a solution containing 10 μL of luciferase, 85 μL of 0.10 M Tris-HCl (pH 8.0) and ATP at final concentrations in the range of 0.02 to 2 mM. Both assays were performed in triplicate. The K_M_ values were calculated using Lineweaver–Burk plots taking the peak of intensity (I_0_) as a measure of V_0_, according to Equation (S1) (Supplemental information). All measurements were done in triplicate and averages were reported. The kinetics of luminescence reaction was performed using a TD-III luminometer (Japan). In the assay for the reaction starting with LH_2_ and ATP, 5 μL of a solution consisting of 80 mM MgSO_4_ and 40 mM ATP were mixed to a solution consisting of 10 μL of luciferase, 10 mM LH_2_ in 85 μL of 0.10 M Tris-HCl buffer pH = 8.0.

### 3.6. Luciferyl-Adenylate Synthesis

The luciferyl-adenylate was prepared using LH_2_ acid and AMP sodium salt (SIGMA) by following a previously described preparation procedure for D-luciferyl-adenylate [46]. Luciferyl-adenylate was analyzed with silica gel TLC (moving phase: ethyl acetate/ethanol/water (5:3:2), followed by revelation by fluorescence with a UV lamp. Luciferyl-adenylate displayed yellowish fluorescence with an R_f_ = 0.68 (R_f(luciferin)_ = 0.87 with greenish fluorescence). A luciferyl-adenylate concentration was estimated from stoichiometric amounts of luciferin and ATP used for its synthesis. According to such estimations, luciferyl-adenylate concentration in stock solution are in the range between 5 to 10 mM.

### 3.7. Determination of k_cat_ and k_ox_

The overall *k*_cat_ and *k*_ox_ were determined according to Equation (S2) (Supplemental information), by calculating the ratio of luminescence activities in *cps* by the number of luciferase molecules based on the specific bioluminescence activities measured with luciferin and ATP (overall *k*_cat_), and with luciferyl-adenylate (*k*_ox_), respectively. Because the absolute value of *cps*, in photons/s, could not be determined, the absolute values of *k*_cat_ and *k*_ox_ in s^−1^ could not be determined, and therefore the values were reported in cps (counts for second). Although these values are not absolute, they can be safely used as relative values of catalytic constants.

### 3.8. 6′-Substituted Amino Analogs

All the 6′-substituted amino luciferin analogs (Figure 1) were synthetized as previously reported [33]. Stock solutions of 10 mM were prepared in DMSO and kept at −20 °C in the dark.

### 3.9. Bioluminescence Spectra

Bioluminescence spectra reported here were recorded in ATTO Lumispectra spectroluminometer (Tokyo, Japan) with cooled CCD camera. For the in vitro bioluminescence recorded using the spectroluminometer, 5.0 µL of luciferases were mixed with 90 µL of 0.10 M Tris-HCl pH 8.0, 5 µL of specific substrate (10 mM d-luciferin; luciferyl-adenylate or 6′-amino-luciferin analogs), and 5 µL of 40 mM ATP/80 mM MgSO_4_. The bioluminescence spectra were measured in triplicate for at least three independent luciferase preparations, and the reported spectra represent the average. The peaks were manually estimated, and the peak variation was ±2.5 nm. Above 620 nm, due to the associated lower energy at higher wavelengths, we assumed peak errors of ±3 nm.

## 4. Concluding Remarks

By using combinatory chemistry with 6′-amino-luciferin analogs and *P. hirtus* red emitting luciferase site-directed mutagenesis, we report the selection of novel brighter FR emitting combinations. The combination of *P. hirtus* R215K red emitting mutant luciferase and N5, stood out for its far-red shifted emission (650 nm) and for the highest bioluminescence activity ever reported among FR emitting luciferin-luciferase systems, displaying more than three times higher bioluminescence activity than with natural LH_2_ at 100 µM concentration, and about 1000 higher activity than with commercial *AkaLumine*. The combination also displayed more sustained and much higher in vivo bioluminescence activity in *E. coli* cells, when compared with the wild-type luciferase with LH_2_. Altogether, these properties make RE-R215K/N5 combination very promising tool for bioluminescence imaging of biological and pathological processes in deep mammalian tissues and organisms.

## Figures and Tables

**Table 1:** Kinetic properties of bioluminescence of *P. hirtus* wild-type and R215K mutant luciferases.

Luciferase	Specific Activity (10^9^ cps/mg) *	Oxidative Activity (10^9^ cps/mg) *	K_M_ ATP (μM)	K_M_ LH_2_ (μM)	K_M_ N5 (μM)	*k*_cat_ LH_2_ (cps)	*k*_cat_ N5 (cps)	*k*_cat_/ K_M_ LH2 (cps M^−1^)	*k*_cat_/ K_M_ N5 (cps M^−1^)	*k*_ox_/ K_M_ ATP (cps M^−1^)	*k*_ox_/ K_M_ LH2 (cps M^−1^)
RE WT	70	65	230	7	0.5	8.3	7.1	1.2	14.2	0.03	1.11
R215K	89	83	50	40	1.0	10	9.4	0.25	9.4	0.19	0.23

* cps: counts per second.

**Table 2:** Comparison of the bioluminescence spectra peaks and activities of *P. hirtus* and mutant luciferases with luciferin, 6′-amino-analogs and AkaLumine.

Analogs	Wavelength (Half-Bandwidth) (nm)		Specific Activity (10^9^ cps/mg) **		*k*_cat_ (10^−6^ cps) **	
	RE WT *	R215K	RE WT	R215K	RE WT	R215K
LH_2_	626 (82)	629 (74)	86.3	104	9.6	11.7
NH_2_-LH	612 (87)	614 (83)	33	31.6	3.7	3.5
Mor	634 (92)	644 (72)	22.9	22.2	2.5	2.4
N5	644 (84)	650 (81)	63.4	81	7.1	9.0
N6	651 (84)	656 (76)	12.4	6.2	1.3	0.6
N7	637 (102)	647 (82)	34.2	8.6	3.8	0.9
AkaLumine	681 (81)	683 (85)	0.9	0.4	0.1	0.05

* The spectra for WT-RE with LH2, NH2-LH, Mor and N5 were previously reported [42]. ** cps: counts per second.

## Supplementary Materials

The following are available online at https://www.mdpi.com/1422-0067/22/1/303/s1.
