# Supplementary material for: A Very Bright Far-Red Bioluminescence Emitting Combination Based on Engineered Railroad Worm Luciferase and 6′-Amino-Analogs for Bioimaging Purposes
Source: Int J Mol Sci. 2020 Dec 30;22(1):303. doi: 10.3390/ijms22010303 (PMC7794784; doi:10.3390/ijms22010303)
Supplement: Supplementary file 1 [file ijms-22-00303-s001.pdf]

Supplemental information

$$I_0 = \frac{Imax\,[S]}{KM + [S]};I_{max} \sim V_{max;}\, I_0 \sim V_0 \qquad (S1)$$

Equation S1. Relationships between luminescence intensity (I) and Velocity (V) used in Michaelis-Mentem equation.

$$I_{max} = \mathrm{QY} \cdot\ k_{cat} \cdot\ [E_t];\ I_0 = \mathrm{QY} \cdot\ kcat \cdot\ [\mathrm{ES}] \qquad (S2)$$

Equation S2. Relationships between Luminescence Intensity, catalytic constant and quantum yield: (Imax) maximum intensity; (I_0) Initial intensity; (QY) Bioluminescence quantum yield; (k_{cat}) catalytic constant; ([Et]) total enzyme concentration; ([ES]) enzyme-substrate complex concentration.

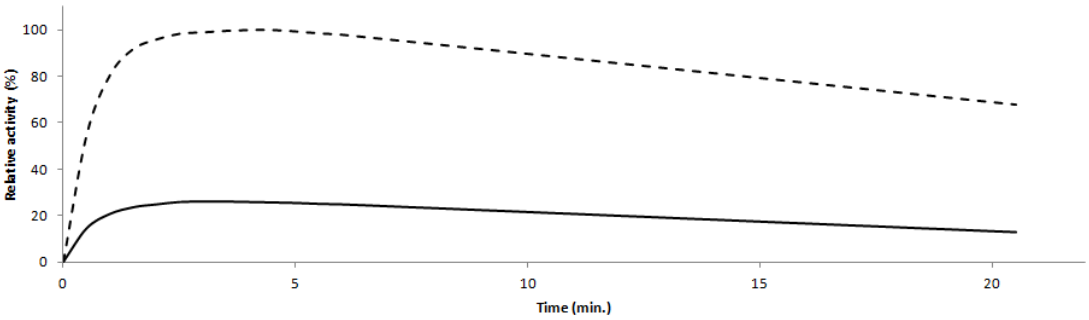

Figure S1. In vivo bioluminescence kinetics of *E.coli* expressing PxRE R215K mutant luciferase: (line) upon addition of 10 μL of 1 mM D-luciferin at pH = 5.0 and (dashed line) upon addition of 10 μL of 1 mM N5 at pH = 5.0.
